# Talking therapy groups on acute psychiatric wards: patients' experience of two structured group formats

**DOI:** 10.1192/pb.bp.114.047274

**Published:** 2016-08

**Authors:** Jonathan Radcliffe, Laura Bird

**Affiliations:** 1 South London and Maudsley NHS Foundation Trust; 2 Surrey and Borders Partnership NHS Foundation Trust

## Abstract

**Aims and method** We report the results of a clinical audit of patients' reactions to two types of talking therapy groups facilitated by assistant psychologists and psychology graduates on three acute wards. Patients' experiences of problem-solving and interpersonal group formats were explored via focus groups and structured interviews with 29 group participants.

**Results** Both group formats generated high satisfaction ratings, with benefits related mostly to generic factors.

**Clinical implications** Adequately trained and supported assistant psychologists and psychology graduates can provide supportive talking groups that patients find helpful.

UK acute psychiatric wards typically have a day or less of weekly input from a qualified psychologist.^1^ Talking therapy groups are a cost-effective method of promoting social interaction and access to psychotherapeutic interventions. In-patient groups are challenging to facilitate – wards can be busy and chaotic, there is high patient turnover, and patients can be disturbed or disruptive in the group.^2^ The evidence base for the outcome of in-patient groups of short duration is limited owing to the difficulties in demonstrating the benefits of brief interventions which are part of a larger, heterogeneous treatment regime and where there are complex patient variables.^3^ However, well-run groups can improve social interaction for patients, many of whom are withdrawn and isolated, as well as providing other benefits derived from talking and thinking.^4^

## Study setting and design

In the current acute psychiatric unit, in-patients can attend groups throughout their admission. Qualified psychological staff facilitate groups such as recovery, social cognition and psychodynamic groups. In addition, assistant psychologists, essentially psychology graduates trained on the job, have facilitated groups for several years. They learn by being apprenticed to experienced colleagues and through fortnightly group supervision. Group facilitation can also be carried out by junior doctors and nursing staff, thus providing valuable training experiences.

The present study focused on two types of groups: a problem-solving format (group 1) and an interpersonal format (group 2), each facilitated by assistant psychologists and psychology graduate volunteers.

### Problem-solving group format

Problem-solving deficits have been found in individuals diagnosed with schizophrenia^5^ and other disorders.^6^ Targeted therapies have been shown to be effective in improving problem-solving skills, remediating verbal problem-solving deficits and helping patients cope with symptoms.^7^ Problem-solving groups can be effective with patients from a range of educational backgrounds.^8^

Grey's^9^ problem-solving format was used on the unit for 4 years, but to our knowledge, it has not been evaluated previously. In this format, hypothetical scenarios of commonly encountered problems are read out to the group by an attendee, and the group then apply three stages of problem-solving: (1) identifying the core aspects of the problem, (2) generating solutions, (3) evaluating these solutions. In addition to improving problem-solving ability, the format aims to provide stimulation and improve social engagement, collaborative working, awareness of others' views and concentration.^10^

Over the preceding year, group 1 (problem-solving format) attendance averaged 5.7 patients per one 1 h session (46 weekly sessions, range 1–9 patients per session, 164 patients attended at least once). Two or three patients attend from each ward, selected after discussions with staff. Patients likely to be highly disruptive are excluded, but otherwise patients who understand English sufficiently can attend.

### Interpersonal group format

Interpersonal theory emphasises the role interpersonal format deficits play in contributing to and maintaining mental health problems. Doerfler *et al*^11^ found 25 to 38% of in-patients had interpersonal format difficulties prior to admission. Interpersonal format groups facilitate patients relating to each other in the ‘here and now’ to help them communicate more clearly, express positive emotions, identify mannerisms that attract or deter others, listen, be supportive, foster disclosure and initiate relationships.^12^

Our interpersonal group uses Hajek's^13^ adaptation of Yalom's^4^ approach for lower functioning in-patients, whereby a topic is introduced to facilitate personal disclosure; for example, ‘Describe an important change in your life’ and ‘Where do you imagine yourself to be in five years?’ Participants then express their feelings and discuss their experiences, give and receive interpersonal feedback. The aims are for patients to engage with each other, reduce isolation and hospital-related anxieties, provide an opportunity to be helpful to others, and help patients realise that they have problems in common, leaving them feeling less alone (‘universality’). For an illustration of interpersonal format group work with in-patients, see Hajek.^14^ Weekly group 2 (interpersonal) attendance at 41 sessions over the year averaged 4.8 patients (range 1–9) and 121 individuals attended at least one group.

## Aims

The aim of this study was to explore patients' experience of two non-qualified therapist-led therapeutic groups in relation to the stated aims of each format (problem-solving or interpersonal format).

## Method

An exploratory, sequential, mixed-methods design was used,^15^ integrating qualitative and quantitative methods. In phase one, qualitative data were gathered in two focus groups. These were used to generate a series of qualitative and quantitative questions to be used in phase two, which consisted of individual interviews (questionnaire available from the authors on request). Quantitative results are reported, together with qualitative data used to offer explanations and illustrate the diversity of views. In the discussion, facilitators' reflections aid a further interpretation of the findings.

### Focus groups

Two focus groups using a standard method^16^ were set up to generate the questions for the individual interviews. Four to five patients participated in each group. The focus groups took place immediately after the therapy groups so participants' experiences were fresh in their minds.

Additional questions for the individual interviews were generated from aims specified in the manuals for each group format.^4,9,13^ The questionnaire consists of 28 closed and open-ended questions and was used for evaluating both group formats, with the exception of a question relating to the three stages of problem-solving, which was omitted for group 2 attendees. One of the authors (L.B.) piloted the questionnaire with five in-patients, after which some questions were deleted and the wording of others improved.

### Individual interview participants

All patients who attended a group over a 4-month period were invited to take part. In all, 29 patients completed 30 semi-structured questionnaires, 15 for each format, and 1 patient completed a questionnaire for both formats; 16 patients had attended both formats, and the rest had attended only one.

### Procedure

Participants were interviewed on the ward by an independent interviewer not involved in the groups, who emphasised confidentiality and wrote down responses. Interviews took 15–20 min. Respondents had attended an average of 1.4 groups of the format in question. They were interviewed up to 3 days after attending the group.

### Analysis of questionnaire responses

Descriptive statistics were applied to closed-question responses and a thematic analysis^17^ to open-ended responses. The thematic analysis followed six phases:^17^ (1) familiarisation with the data; (2) generating initial codes; (3) searching for themes; (4) reviewing potential themes; (5) defining and naming themes; and (6) producing the report. As similar ideas were shared across the various open questions, the coded responses were themed together where similar ideas were being communicated. Another investigator (J.R.) checked coding and themes for credibility.

## Results

### Generic benefits

Overall satisfaction rates were high as shown by closed-ended questions (Table 1).

**Table 1:** Satisfaction rates for problem-solving (group 1) and interpersonal (group 2) formats based on the number of positive (Yes) responses in both groups

Did you …	Group 1 *n* = 15	Group 2 *n* = 15
Enjoy the group?	14	15

Feel the group was supportive?	14	13

Find using the task difficult?	3	5

Find the group helpful?	15	13

Find the structure of the group useful?	13	13

Find the task helped you talk about your own experiences?	11	9

Find the group relevant to you?	10	12

Find out useful practical information within the group?	8	9

Find it helped you feel more able to cope with your own challenges?	10	13

When asked what aspects of the group the participants liked or found helpful, several referred to the value of doing something active off the ward (group 1 (problem-solving format), *n*=6; group 2 (interpersonal format), *n*=1). Six group 1 attendees enjoyed being with others in a group, whereas six group 2 attendees valued the opportunity to talk freely with others in a supportive environment. With regard to the aspects of the groups the participants disliked or found unhelpful, more group 2 (*n*=8) than group 1 (*n*=1) patients referred to other patients' behaviour, for example talking too much or too little, interrupting and walking out of the group.

Respondents from both groups thought their groups worked well together (group 1, *n*=14; group 2, *n*=10) and helped them to concentrate (group 2, *n*=13; group 1, *n*=14). Both formats aimed to help patients become more aware of others' viewpoints, but more group 1 respondents thought it was useful to hear other patients' views than group 2 respondents (*n*=15 and *n*=10 respectively). When asked why this was important, group 2 responses were more interpersonally oriented, for example, ‘I have realised that I am not the only one with problems' and ‘It is useful to hear somebody else speak about what they think – not feeling different or alone’.

Although the interviews involved questions about the group that patients had attended most recently, nine participants had also attended the other type of group. Of these, five preferred the problem-solving format (of group 1) and four the interpersonal format (of group 2).

### Problem-solving skills

When asked whether they had developed problem-solving skills, unsurprisingly, more group 1 respondents felt they had done so (*n*=11; group 2, *n*=4). When asked how the group helped them achieve that, group 1 members said focusing on a scenario helped, for example ‘It made me think more and sort things out’, ‘It showed how I can deal with a situation if I put my mind to it’. Although most group 1 patients felt that they had learned to solve problems better, only one could name the three stages of problem-solving used in the group; six recalled one or two stages.

### Interpersonal learning

A key aim in an interpersonal group format is for patients to learn more about themselves. Participants in both groups were asked whether they had learned anything new about themselves, and there were no differences between the groups, with nine participants in each group saying ‘yes’. When asked the follow-up question, group 1 attendees tended to make general affirmative statements such as ‘I like people’, ‘I am able to communicate with people, I can express myself’ and ‘I am more intelligent than I thought I was’, whereas group 2 attendees’ responses included ‘I learned to stay calm and listen to others’, ‘I was able to be more open in front of others and express myself more without getting anxious’ and ‘[I was able to] get on with other people and how to listen to them’.

Another aim of the interpersonal group format is to facilitate disclosure of personal information and feelings. There were no differences between the groups in this respect, however, some differences were revealed by the follow-up qualitative question. Group 1 attendees expressed their views in relation to solving the problem (*n*=6 *v. n*=0 in group 2), whereas group 2 respondents said that they had talked about personal or emotional experiences (*n*=8 *v. n*=1 in group 1), for example, ‘I shared my experience of bipolar [disorder]’ and ‘How I deal with anger or don't’. Moreover, attendees said it was good to discover that others had similar experiences and problems. These experiences correspond to Yalom's interpersonal format aim of ‘universality’ (*n*=4; group 1: *n*=1).

## Discussion

The Department of Health emphasises the value of groups with psychological focus.^18^ Researchers have demonstrated the benefits of having a varied therapeutic programme containing different types of group.^19^ Yalom^4^ describes the generic benefits of in-patient groups common to a range of groups as well as benefits that are specific to each particular type. Yalom's generic benefits include engaging patients in a therapeutic process, discovering that talking helps, identifying problems, reducing isolation, being helpful to others and reducing hospital-related anxiety. There are few studies that compare patients' experience of different kinds of group, and none that separate generic from specific benefits. The present study was not an outcome study, but an exploratory investigation of patients' experiences of these benefits.

The main limitations of this project were the constraints imposed by the type of data and the small sample size, which meant that inferential statistics were not possible. Moreover, the individual interview questions were somewhat leading. Scaled responses would have improved the design. Nevertheless, several findings that may be relevant for clinical practice have emerged.

The key result was that nearly all attendees were satisfied with the groups. The cited benefits of group attendance were primarily ‘generic’, although there were indications of specific format benefits. The generic benefits included sharing experiences, patients becoming more aware of others' points of view, expressing themselves, improved concentration, learning something new about themselves and hearing others' views. These are consistent with Yalom's generic aims of engaging clients in the therapeutic process and combating social isolation. The results support the notion that ward groups improve morale.^20^ Approximately equal numbers preferred each format showing the value of variety of format. The findings of specific benefits demonstrate the value of using different formats.^19^

An unexpected benefit of the problem-solving format was patients disclosing information about themselves and becoming more aware of others' points of view. Despite these being interpersonal format-specific aims, this suggests these are generic benefits. However, interpersonal group respondents tended to describe sharing personal information rather than sharing their ideas.

Even though problem-solving group attendees could not identify the problem-solving stages, they said that the group helped them think about problems. It is possible that identifying elements of problematic situations, thinking creatively, evaluating solutions and using language to express these is valuable, especially when problems relate to current frustrations. Going through each stage may teach patients to think more logically and sequentially about problems. However, this needs evaluating.

More patients complained about other patients' behaviour in the interpersonal format group. Qualitative data suggested that attendees found the interpersonal format group more challenging and felt more contained in the problem-solving group. This is consistent with the second author's experience of the problem-solving group, which provided a more tightly imposed structure, making it especially suitable for patients with anxiety, although facilitators tend to prefer the interpersonal format group as it is less repetitive to facilitate. Disclosing personal feelings can make some patients feel anxious. On the other hand, comments showed that patients especially valued sharing more personal information in a supportive environment. The facilitators' view is that when the interpersonal format group works well, patients find sharing their experiences satisfying and this creates a sense of togetherness. In both formats, the facilitators set ground rules that patients should respect each others' opinions and give each other a chance to speak. In the more open agenda of the interpersonal format group, facilitators sometimes find it difficult to ensure everybody gets to speak, particularly if a group comprises more vocal patients or those who are less assertive. Patients deviate from the ground rules more in the interpersonal format group with its more fluid structure. The problem-solving group has the advantage in terms of the facilitator being able to direct the group to the task. Although Yalom describes interpersonal format group difficulties that emerge during a session as ‘grist for the mill’, it is difficult for less experienced staff to use the ‘grist’ therapeutically. Facilitating and handling social feedback requires more skill, for example when patients criticise others and anger or distress each other. Overall, interpersonal format groups may provide deeper benefits but require more skill and longer time shadowing an experienced practitioner. The more structured problem-solving groups may be more suitable for less experienced staff. Further comparative research is needed.

The phased mixed-methods approach gave validity as the individual interview questions were partly derived from the focus groups. Methodology could be improved in future studies through more detailed analysis of focus groups transcripts which would produce a richer picture of themes and differences. Individual interviews similarly would have greater empirical precision with more cautious avoidance of leading questions. Mean age of interviewees, gender make-up, diagnoses, length of stay would add value, as well as control for number and type of groups previously attended. A third non-talking group could be added to provide further basis for exploration of generic benefits of groups, such as a relaxation group.

To conclude, the study found good satisfaction with groups, showing that suitably trained, less qualified staff can provide valuable experiences for in-patients as part of a therapeutic programme. At a time when resources are limited, further research could explore whether it is primarily the process of taking part in groups that benefits patients, how much the format of the group matters, and which core principles make an intervention valuable. With respect to facilitation of groups, future research could compare the core skills and attributes needed to run different types of groups, and the efficacy of groups facilitated by less qualified staff such as graduate volunteers or peer support workers.
